# Practice makes better? The influence of increased practice on task conflict in the Stroop task

**DOI:** 10.3758/s13421-024-01677-7

**Published:** 2025-01-25

**Authors:** Jonathan Najenson, Rut Zaks-Ohayon, Joseph Tzelgov, Nir Fresco

**Affiliations:** 1 Department of Philosophy, Ruhr-Universität Bochum, 44780 Bochum, Germany; 2 Mental Health Institute of Beer-Sheva, Ministry of Health, Beer-Sheva, Israel; 3 Faculty of Health Sciences, Ben-Gurion University of the Negev, 84105 Beer-Sheva, Israel; 4 Department of Cognitive and Brain Sciences, and School of Brain Sciences and Cognition , Ben-Gurion University of the Negev, 84105 Beer-Sheva, Israel

**Keywords:** Practice, Cognitive control, Task conflict, Stroop task, Automaticity

## Abstract

The Stroop task is widely used to study attentional control and cognitive flexibility. However, questions about its sensitivity to training and the impact of task conflict on attentional control remain open. We investigated the effects of practice and task conflict on attentional control in the Stroop task, with participants completing four sessions of a Stroop task over 3 weeks in low and high task-conflict conditions. Our results show that the level of task conflict had an impact only in the first session, even though participants remained sensitive to task conflict throughout all four sessions. Moreover, we found that practice reduced response times in the Stroop task, for both congruent and incongruent trials. Nevertheless, the interference between congruent and incongruent stimuli remained consistent over the 3-week period, indicating that inter-condition interference is not affected by training. Our study, therefore, suggests that the extent to which the level of task conflict modulates Stroop task performance is only partially sensitive to training. These findings provide further insights into the role of task conflict and practice in attentional control and cognitive flexibility.

## Introduction

Skilled action involves a delicate balance between maintaining task goals and resource-efficient automatic responses. Striking the right balance between these two opposing demands requires cognitive control mechanisms for applying strategies relevant to current task contexts, as well as automatic response patterns. Practice plays a crucial role in reaching a balanced task execution, because it can either shift towards increased automaticity, resulting in a more efficient performance, or foster greater cognitive control, with the possibility of interference from irrelevant information. Understanding how practice contributes to developing this balance is, thus, important for the study of skilled action.

Repeated practice has long been known to influence the balance between automatic and controlled behavior (Cohen et al., 1990; Haith & Krakauer, 2018; Logan, 1979; MacLeod & MacDonald, 2000; Posner, 1975; Moors & De Houwer, 2006; Yang et al., 2022). The interplay between control and automatic behavior that leads to skill acquisition appears to exhibit a temporal dynamic (Tenison & Anderson 2016). Earlier performance is more intentionally guided compared to later stages of training at which habitual responses are more frequent (Logan, 1988). One of the main factors associated with automatic performance is the processing speed of task execution (Logan, 1985; Moors, 2016). The increased familiarity with a specific task results in shorter response times (RTs), a pattern that is consistent with the “law of practice” (Anderson, 1981). The decrease in response latency is considered a central marker of the automatization of task performance (Hardwick et al., 2019; Logan, 2002; Moors & De Houwer, 2006). Through repeated practice, the balance between automatic and cognitively controlled behavior changes, with automatic tasks demonstrating shorter RTs and controlled tasks exhibiting longer RTs.

This balance has been investigated by examining how automatic responses interfere with controlled behavior. The Stroop task is used to probe cognitive control by measuring the ability of participants to attend to task-relevant information and to inhibit automatic responses to task-irrelevant information. In the classic Stroop task, participants are presented with color words and instructed to (a) identify the color of the stimuli, and (b) ignore the (meaning of the) word. Participants typically respond slower when the word and color are inconsistent (e.g., when GREEN is presented in blue font) than when they are consistent (e.g., when BLUE is presented in blue font). This effect – dubbed the *Stroop interference effect* – shows the influence of automatic processing on task performance (Dyer, 1973). The Stroop effect, thus, offers a window into the dynamics of the balance between cognitively controlled and automatic processes in response to conflicting information (Besner et al., 1997).

Understanding how training influences automaticity is one of the three major issues Macleod (1991) designated as being crucial to discovering what causes the Stroop effect. The fact that reading is a significantly practiced skill results in participants responding to the word stimuli despite an opposing task demand to only identify the colors. This long practice prevents participants from completely suppressing the task-irrelevant stimuli, which interferes with the identification of colors. The difference in the amount of practice participants have had with reading words raises the question of whether this amount of practice is the source of observed interference.

Current accounts emphasizing the role of learning in driving Stroop task-related effects have focused exclusively on frequency (i.e., a larger proportion of incongruent items in the list; Bugg et al., 2011) or recency (i.e., transition between congruent/incongruent trials; Kane & Eagle, 2003). Practice effects, however, remain largely underexplored. Congruency effects have long been known to decrease with practice (Dulaney & Rogers, 1994; Dulaney & Ellis, 1991; Macleod, 1998). Macleod (1998), for example, trained participants on a version of the Stroop task for five and ten consecutive days and showed that interference from incongruent stimuli was significantly reduced over time. Nevertheless, interference was not *eliminated* even after considerable training.

Previous studies employing longer practice trials to examine congruence effects did not separate between different types of conflict that may cause interference. Different types of conflict can influence performance in the Stroop task (Levin & Tzelgov, 2014; Littman et al., 2019). Two established types of conflict are (a) informational conflict, and (b) task conflict. *Informational conflict* derives from performing tasks with opposing demands. While participants are asked to identify the stimulus’ color, they also read the word, thereby being exposed to contradicting informational signals (e.g., the semantic information of the irrelevant word conflicts with the sensory information of the relevant color), each demanding a different response.

*Task conflict*, on the other hand, refers to a competition between two possible task sets and reflects the suppression of automatically activated word-reading processes when participants are instructed to identify colors (MacLeod & MacDonald, 2000). Since word reading is a more familiar task than color identification, a conflict arises already at the *preparatory* stage (i.e., when participants are provided with instructions about the task) before the two dimensions of the Stroop stimuli compete, and, thus, produce an informational conflict (Braverman & Meiran, 2015; Monsell et al., 2001; Steinhauser & Hübner, 2009). A key method of modulating task conflict in the Stroop task is to vary the proportion of non-word color stimuli in the list (i.e., neutral stimuli, such as ‘#@*’).^1^ Since words interfere more with color identification than non-words (Levin & Tzelgov 2016), they result in shorter RTs in the neutral trials. Tasks in which there is a higher proportion of neutrals display larger negative facilitation (i.e., shorter RTs in the neutral than in the congruent condition), an effect termed the *negative facilitation effect* (Entel et al., 2015, 2018). Task conflict and the negative facilitation effect are arguably related: a low level of task conflict (i.e., majority of neutrals) is necessary for demonstrating the negative facilitation effect.^2^

Prior research on the influence of practice on Stroop-like interference has not distinguished between informational and task conflict, leaving unresolved the question of how much practice influences congruency effects at different levels of task conflict. Whether adjusting varying proportions of conflicting information can change one’s ability to selectively attend to task-relevant information, while suppressing automatic task-irrelevant responses, remains an important open question. The present study provides a step in this direction. To date, prior studies have not examined how learning changes over longer time periods. Specifically, they have not examined how practice changes one’s ability to selectively attend to task-relevant information, while suppressing automatic task-irrelevant responses given varying proportions of conflicting information.

Nevertheless, the ability to selectively attend to task-relevant information is an important aspect of skillful behavior that can be enhanced through extensive practice (Moors, 2016). Practice typically entails that one becomes more familiar with the structure of a task set (Steyvers et al., 2019; Yeung & Monsell, 2003). Previous research has shown that prior experience with conflict influences subsequent cognitive control adjustments (Gratton et al., 1992). Additionally, repeated exposure to high-conflict situations is believed to engage cognitive control mechanisms (Botvinick et al., 2001). Therefore, it is reasonable to assume that the congruency effect would be reduced after more trials with a lower proportion of neutral stimuli, as participants would be primed to expect a high level of conflict in subsequent trials and would adapt their attentional control accordingly. Hence, increased practice with a task involving a high level of task-conflict should result in an improved attentional ability, thereby reducing interference in the Stroop task – where word and color are simultaneously presented (Melara & Algom, 2003). This can be likened to a "Learning under Fire" context. In a high-conflict stimulus environment, the ability to selectively attend to task-relevant information, while suppressing automatic task-irrelevant responses, would improve as more cognitive resources are allocated to handle the task.

Moreover, it is well established that attentional allocation is sensitive to the cognitive demands imposed by the environment (Lavie, 2010; Mackie et al., 2013). We anticipated that interference would diminish over time when task conflict is high, especially when participants had already practiced the task set. After a given number of trials, participants would expect a significant presence of distractors and adjust the capacity of their attentional mechanisms to meet the cognitive demands imposed by the task structure.

Although prior studies have examined the effects of task conflict on attentional allocation, they have not fully investigated how the way attentional allocation is structured within a task changes with practice. More specifically, they have not investigated how task conflict influences these changes. To fully comprehend the impact of task conflict on the ability to attend to task-relevant information, while inhibiting task-irrelevant responses, it is important to examine changes in attentional allocation in both single- and multiple-session tasks. Doing so enables controlling for the influence of task conflict on expected stimuli in a task set over time.

The present study, thus, aimed to examine the extent to which conflict modulates learning over time by manipulating high and low task-conflict conditions across multiple sessions. To test our hypothesis, we varied the proportion of neutral stimuli and administered four sessions of the Stroop task over a period of 3 weeks. We predicted that the level of task conflict would significantly influence performance in the Stroop task, such that participants in the high task conflict group would reduce RTs across congruent and incongruent conditions compared to participants in the low task conflict group. Following previous studies (Macleod, 1998), we predicted that the delta in RT between congruent and incongruent conditions, as represented by a negative slope, would decrease over the course of multiple sessions.

## Methods

### Participants

The study was preregistered in the Open Science Framework (OSF) archive before it was conducted (see https://archive.org/details/osf-registrations-hq7d3-v1). Thirty-two students from Ben-Gurion University of the Negev (23 female) participated in the experiment in partial fulfillment of their course requirements. (Prior to conducting the experiment, approval was obtained from the ethics committee.) All participants were native Hebrew speakers, had normal or corrected-to-normal eyesight, and did not suffer from any learning disabilities or attention deficits. To determine our sample size, we conducted an a priori power analysis using MorePower software version 6.0.4 (Campbell & Thompson, 2012). The analysis revealed that a sample size of 16 participants per group was sufficient to observe the effect in which we were interested – the three-way interaction between group, session, and congruency (α = .05, with an effect size of η2p = .55, and a power of .85). The estimation of the effect size of the interaction was based on prior research with similar designs (Hennessey et al., 2014; Jostman & Koole, 2007; Milliken et al., 2012; Shichel & Tzelgov, 2018).

### Stimuli and apparatus

We used a Hebrew Stroop task with four colors: red, green, blue, and yellow. The congruent stimuli were generated by printing each of the color names in its own color (e.g., the Hebrew word for red (אדום) was printed in red), resulting in four congruent stimuli types. The incongruent stimuli were generated by printing each word in all four colors excluding its own (e.g., the Hebrew word for red (אדום) was printed in yellow, green, and blue), thereby resulting in 12 incongruent stimuli types. Additionally, we used the pattern #### as a non-word neutral stimulus, which we generated in the four colors (i.e., the pattern #### was printed in red, blue, green, and yellow), thereby resulting in four different neutral pair types. Note that the hebrew words for all colors used in our design are four-lettered.

The stimuli were presented on a widescreen Dell LED monitor (U2722D, color profile D6500) with a resolution of 1,920 × 1,080, in boldfaced 22-point Times New Roman font. Data collection and stimuli presentation were controlled by E-Prime 2.0 software (Psychology Software Tools, Pittsburgh, PA, USA) on a Dell computer (OptiPlex 7070) with an Intel Core i7-9700 processor 3.0GHz, 32GB RAM memory. The three types of stimuli (congruent, incongruent, and neutral) were randomly ordered throughout the task.

### Design and procedure

Participants were randomly assigned to one of two groups that differed by the words-to-neutrals ratio. The first group, which is referred to hereafter as a "high control" group, was exposed to 80% word stimuli trials (480 trials: 240 congruent and 240 incongruent), and 20% neutral stimuli trials (120 trials). The other group, which is referred to hereafter as the "low control" group, was exposed to 20% word trials (120 trials: 60 congruent and 60 incongruent) and 80% neutrals (480 trials).

The experiment structure is as follows. It started with task instructions, which guided participants to ignore the meaning of the written word and only respond to the color in which it is printed – by pressing a button of the same color as the printed word. Then, a 20-trial practice was performed to ensure that participants understood task instructions. Following the practice, participants were informed that the practice was over and were asked to press the space bar to proceed to the experimental trials.

The stimuli structure of every trial is as follows. Each trial started with a 250-ms fixation cross in the middle of the screen, followed by a target slide in which the stimuli were presented for 1,000 ms, and ended with a blank screen for an interval of 500 ms between trials. Participants were allowed to respond only as long as the target slide was presented. Each participant responded to 600 trials in total.

The overall time span of the experiment was 3 weeks. The second session was conducted 24–48 h after the first session. The third session was conducted 6–7 days after the second session. The fourth session was conducted 12–14 days after the third session. Each participant participated in four identical sessions of the task.

## Results

Mean RTs of correct responses over 350 ms (in 99% of the data) were analyzed using a three-way repeated-measures analysis of variance (ANOVA) with group (high control and low control) as a between-participant variable, congruency (congruent, incongruent, and neutral) and session number (1–4) as within-participant variables. A post hoc power analysis revealed that our experimental design has 85% power to detect effects of ηp2 = 0.048, meaning that our design is well powered to detect all effects reported in this section.

Using the classical approach to calculating speed-accuracy trade-off effects, we first calculated the RTs and error percentages separately, then verified that the effects were not reversed (see, e.g., Sorensen & Woltz, 2015). To further investigate the possibility of a speed-accuracy trade-off, we calculated the multilevel correlation between RT and accuracy. A small but significant positive correlation was found, r = 0.10, p < .001, indicating that trials with longer RTs were slightly more accurate. While this trade-off effect is small, we conducted additional analyses to determine its source. A hierarchical linear model (HLM) was built, predicting RT from accuracy and congruency, with accuracy within subjects as a random effect. The analysis revealed no main effect of accuracy, F(1, 25) = 1.24, p = .27. However, the two-way interaction between accuracy and congruency was significant, F(2, 4633) = 5.50, p = .004, η_p_^2^ = .002. Follow-up analyses revealed that that the speed-accuracy trade-off arose entirely from the neutral condition. Indeed, when the analysis was conducted without the neutral condition, the speed-accuracy trade-off was no longer present, r = 0.007, p = .16. Taken together, these results indicate that the speed-accuracy trade-off does not account for the main findings of our analysis.

**Fig. 1:**
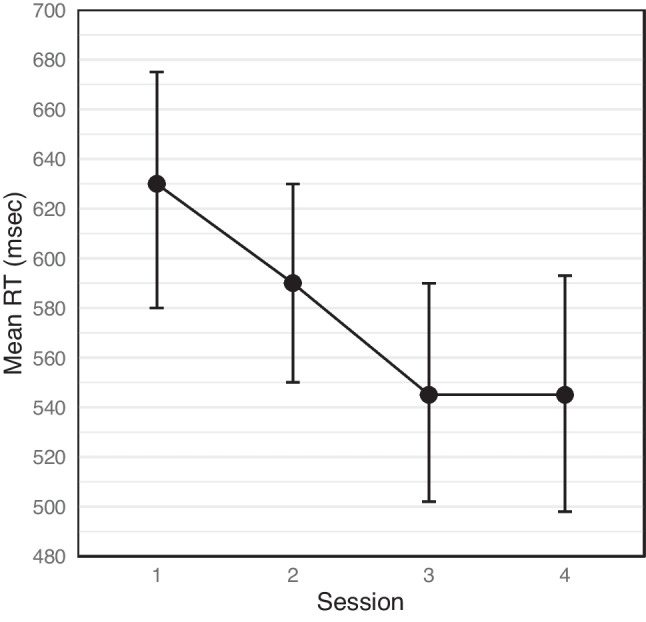
Mean reaction times (RTs) across sessions in the Stroop task, averaged across high and low control groups and for all conditions (congruent, incongruent, and neutral) combined. Bars represent standard errors

Our main analysis revealed a significant main effect for session (see Fig. 1), F (3,90) = 73.58, MSE = 1990, p < .001, η_p_^2^ = .71. To further examine the effect of practice time (which is reflected in the session variable), we conducted a linear trend analysis of the effect, F (1,30) = 97.44, MSE = 3499, p < .001, η_p_^2^= .76, which revealed a decrease in the RT as a function of the session number. In addition to the overall decrease in RT, we also examined the delta values, which represent the difference in mean RTs between congruent and incongruent conditions for each group and session. The delta values for the High control group were 38 ms, 22 ms, 20 ms, and 18 ms for sessions 1, 2, 3, and 4, respectively. For the Low control group, the delta values were 76 ms, 38 ms, 47 ms, and 24 ms for sessions 1, 2, 3, and 4, respectively. These results indicate that the difference in RTs between congruent and incongruent conditions generally decreased over the course of multiple sessions.

**Fig. 2:**
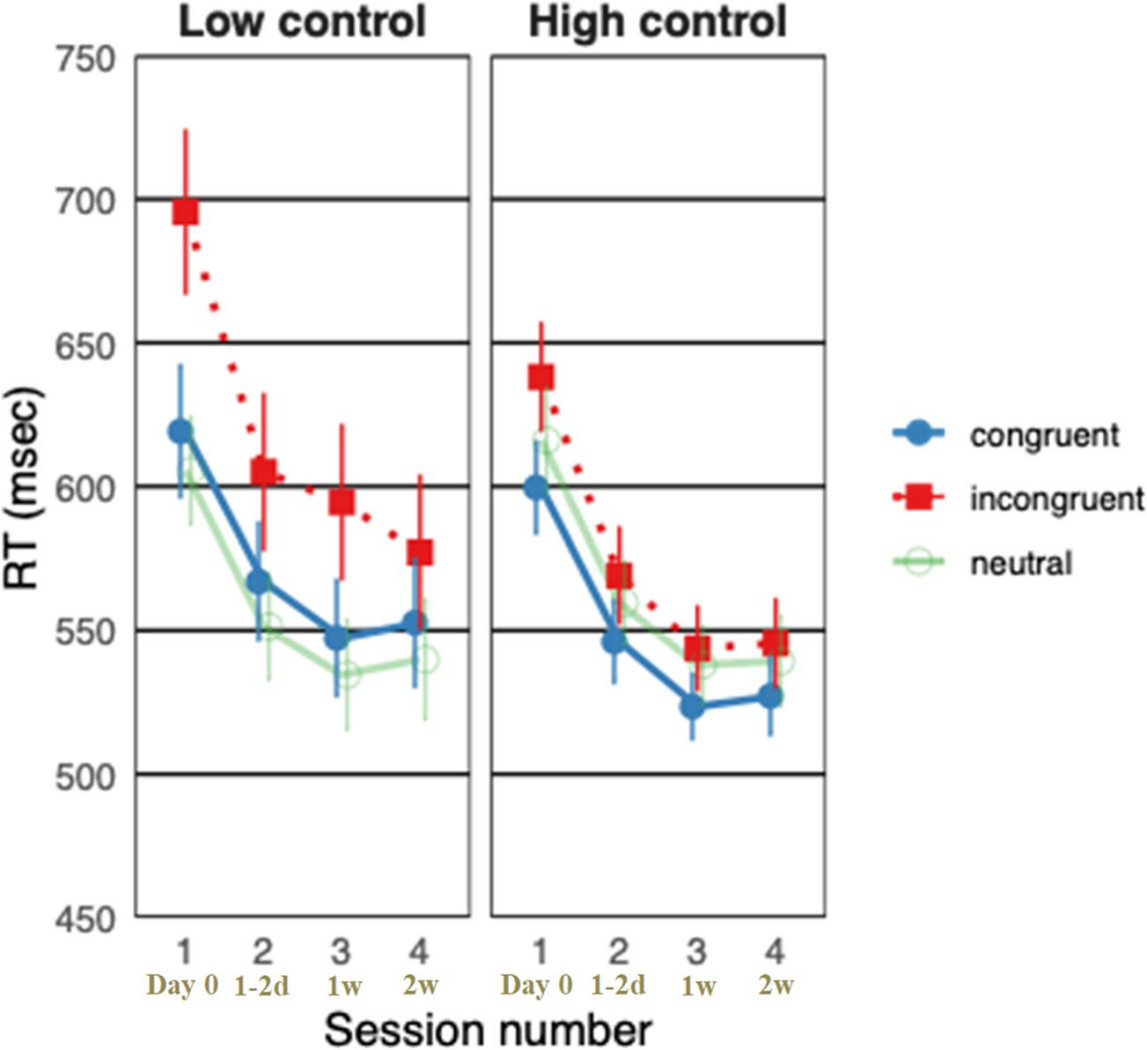
Comparison of mean reaction times (RTs) for congruent, incongruent, and neutral conditions across four sessions under low and high task conflict conditions. Bars represent standard errors. Time stamps below session numbers indicate the time of each session relative to the previous session (Day 0 = initial session, 1–2d = 1–2 days after initial session, 1w = 1 week after the second session, 2w = 2 weeks after the third session)

A main effect for congruency was found as expected (see Fig. 2), F (2,60) = 55.85, MSE= 1073, p < .001, η_p_^2^ = .65, resulting in significantly longer RTs for incongruent trials (596 ms), and no significant difference between congruent (559 ms) and neutral (560 ms) trials. In addition, the two-way interaction between group and congruency, F (2,60) = 21.097, MSE = 1073, p < .001, η_p_^2^=0.42, was significant, as well as the two-way interaction between congruency and session, F (6,180) = 10.252, MSE = 241, p < .001, η_p_^2^ = .25. To examine practice effects in each participant, we performed an additional analysis with subject as a factor. The results revealed no significant difference between participants, F (1, 29) = 1.314, MSE = 69795, p = .261, η_p_^2^ = .04, suggesting that the congruency effect's sensitivity to practice is consistent across individuals in our study.

Importantly, our analysis revealed a three-way interaction between group, congruency, and session (see Fig. 2), F(6, 180) = 2.56, MSE = 241, p = .021, η_p_^2^ = 0.08. We conducted a follow-up analysis to investigate how the group × congruency interaction evolved across the four sessions. A significant group × congruency interaction was found within each session, indicating that the Stroop effect differed between the high control and low control groups throughout the experiment (i.e., the difference between congruent and incongruent conditions). Table 1 shows how the Stroop effect within each group changes with sessions. As shown in Table 1 and as the linear trend analysis indicates, there was an overall decrease in RTs observed across sessions, with the largest change in the Stroop effect occurring between the first and second sessions, particularly for the low control group.

**Table 1:** Group × Congruency interaction within each session. This analysis resulted in four comparisons of the Group × Congruency interaction (one for each session)

Session	F (2,29)	p-value	High control Stroop effect (ms)	Low control Stroop effect (ms)	Difference in Stroop effects (ms)	Std. Error	η_p_^2^
1	10.584	**< .001**	35	77	42	25.1	.42
2	13.593	**< .001**	21	35	14	21.1	.48
3	11.169	**< .001**	17	45	28	21.6	.43
4	10.507	**< .001**	16	24	8	21.7	.42

*Note*: All p values < 0.01 even when adjusting for multiple comparisons

Comparing the two-way interactions between the sessions using orthogonal planned contrasts (weights of [−3, 1, 1, 1] for session number, [−1, 1] for group, and [−2, 1, 1] for congruency) suggested that the difference in Stroop effects was larger for the first session compared with the rest, F(1, 30) = 6.13, MSE = 408, p = .018 (uncorrected), η_p_^2^ = 0.17 (see footnote for consideration of correction to multiple comparisons).^3^ When we conducted the two supplementing orthogonal comparisons, examining the difference in Stroop effects between the second session with the third and fourth (weights of [0, −2, 1, 1] for session number), and the difference in Stroop effects between the third and fourth (weights of [0, 0, −1, 1] for session number), the effects were not significant, F(1, 30) = 0.02, MSE = 323, p = .873, η_p_^2^ = 0.06 and F(1, 30) = 3.94, MSE = 282, p = .063, η_p_^2^ = 0.11, respectively. Combined, these results suggest that the three-way interaction stems from the difference in Stroop effects being significantly larger in the first session compared with the others, while from the second session onwards, there is no longer a significant decrease in the difference in Stroop effects.

**Fig. 3:**
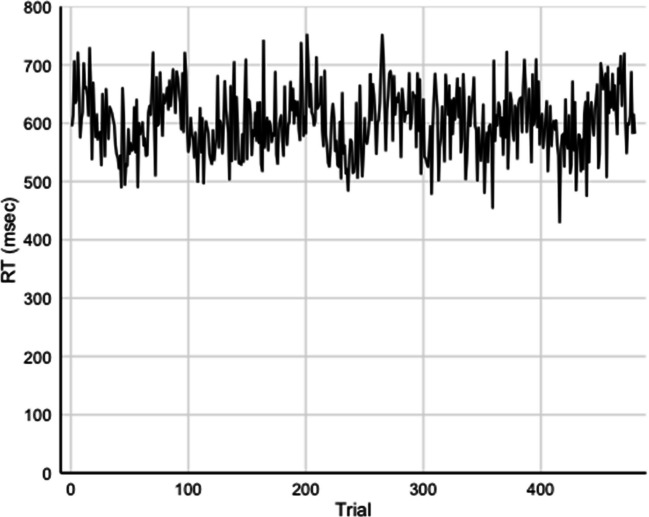
Individual trial reaction times across congruent and incongruent trials in session 1 displaying the natural trial-by-trial variability in participant performance

To explore potential changes in performance within the first session, we conducted an analysis dividing this session into two halves. We examined changes in performance based on trial number, treating each trial as a step. The analysis revealed no significant change in performance across the session, F(1, 467) = 1.054, MSE = 625, p = .15, η_p_^2^ = 0.002, indicating that participants' performance remained relatively stable throughout the first session (see Fig. 3).

A Bayesian analysis of our data suggests that the model incorporating the three-way ANOVA interaction between session, congruency, and group is the most likely, given the distribution of our results. The likelihood of the hypothesis of our model over the null has a BF10 = 2.976E+70. When comparing alternative models, which included different combinations of factors and their interactions, the three-way ANOVA model outperformed all others, including main effects models (e.g., session: BF10 = 4.021E+49) and two-way ANOVA models (e.g., session + congruency: BF10 = 5.310E+66). Our results indicate decisive evidence in favor of this model over the null model and all other alternative models, suggesting that the interaction between session, congruency, and group best explains the observed data. An increase of the value of the BF reflects an increase in the chance of one hypothesis over the other.^4^ Given that the three-way ANOVA resulted in a very small effect size, and the variance found – following an analysis of the data as a function of session – resulted mostly from the first session, we expected the two-way ANOVA model to have a higher BF10 than the three-way ANOVA model. This further supports the importance of the three-way interaction in explaining the observed data.

## Discussion

In the present study, we found that the level of task conflict is inconsequential with repeated practice. Although participants remained sensitive to task conflict throughout all sessions, the influence of task conflict on practice-related improvements was limited to the first session. After the first session, performance gains were similar for both high and low task-conflict conditions, suggesting that task conflict's impact on learning in the Stroop task is most significant during initial exposure (see Fig. 2). Additionally, our findings confirm the presence of the practice congruency effect, as observed in prior research, demonstrating a reduction in sensitivity to interference with increased practice. However, this effect was limited to intra-condition performance and did not transfer to inter-condition performance.

### The influence of task conflict following practice

The findings of this study show that while the level of task conflict continued to affect RTs in the Stroop task across all sessions, its influence on practice-related improvements was limited to the first session. While previous studies showed a robust difference in RTs when the proportion of neutral stimuli varies (Bugg et al., 2011; Kane & Engle, 2003; Lindsay & Jacoby, 1994; Shichel & Tzelgov, 2018), in our study, the task conflict effect was not present following extended training. We found an interaction effect, indicating that the presence of task conflict influenced performance only in the first session. From the second session onward (24–48 h after the first session), the different proportion of neutral stimuli was not driving the decreased patterns in RTs. This finding contrasts with our expectations that practice, defined here as the number of trials over time, would benefit from a high-task conflict.

Considering that task conflict already arises at the preparatory stage (when participants are provided with the task instructions; Goldfarb & Henik, 2007), the reduced influence of task conflict on practice-related improvements in subsequent sessions may express the fact that the influence of whichever cognitive resources that may be available for improvement in task conflict is exhausted in the first session. Adjusting the capacity of attentional mechanisms to meet the cognitive demands imposed by the task structure may rapidly deplete the available cognitive resources, thereby resulting in all feasible improvements being reached within this first session. Therefore, the ability to selectively attend to task-relevant information, while suppressing automatic task-irrelevant responses, does indeed improve in a high-conflict stimulus environment. However, once a plateau effect is reached during the first session, further practice in a high-task conflict environment might not yield additional performance improvements.

Another possibility, which is more consistent with the common interpretation of conflict in the Stroop task, is that participants have learned the structure of the task. Specifically, participants already learn the task sets, which they expect to encounter in later sessions, in the first session. As such, the anticipatory control that influences performance in the first session becomes irrelevant once they have learned the nature of the conflict that is built into the task. Following this line of reasoning, task conflict becomes available for attentional control after participants have learned the task sets.

Recently, it has been proposed that the influence of task conflict on performance might result from the degree of proactive control that participants employ before seeing the Stroop stimulus (Kalanthroff et al., 2018). Accordingly, following the learning of task sets, participants might lower the degree of proactive control, because their allocation of attention is already determined prior to the beginning of the task’s second session.

Proactive control and its relationship to task conflict may be understood within the context of the conflict-monitoring framework. According to this framework, the Stroop effect arises from the conflict between two types of stimuli that recruit cognitive control mechanisms (e.g., Goldfarb & Henik, 2007; MacLeod & MacDonald, 2000; Pardo et al., 1990). The presence and amount of conflict are necessary to recruit cognitive control to modulate or inhibit habitual responses (Blais et al., 2007; Botvinick et al., 2001). A dedicated module, assumed to reside in the anterior cingulate cortex, is responsible for detecting conflicts and initiating control by adjusting top-down attentional selection (Botvinick et al., 2004; De Pisapia & Braver, 2006; Sohn et al., 2007). Task conflict occurs when stimulus-driven behaviors are incompatible with the current goal-directed task and a cognitive control mechanism is recruited to resolve this conflict by biasing attention towards the relevant task goal (Kalanthroff et al., 2018).

However, the absence of practice-related sensitivity to task conflict in the Stroop task beyond the first session raises various concerns about the role of task conflict. The fact that the influence of task conflict appeared primarily in the first session suggests that participants did not rely on a conflict signal after learning the task sets. The task conflict being confined to the first session suggests that the recruitment of a control function by a task conflict signal (Braem et al., 2019) becomes redundant after an engagement with a conflict task. Such redundancy might suggest that a task conflict signal rather has to do with the anticipation of novel conflict and not with the conflict inherent to a task (i.e., a novel conflict indicates a new, different task set).

### The influence of practice on the congruency effect

In line with our predictions, and previous studies, we found that practice reduces RTs in the Stroop task, even relatively to an extensively practiced skill. A general decrease in RTs across all conditions (congruent, incongruent, and neutral) was found in both low- and high-task conflict groups (see Fig. 1). A long-standing question about the Stroop task concerns the task's sensitivity to training. Our findings suggest that RTs in the Stroop task can improve with extensive practice, even in the incongruent condition in which the identity of the stimuli induces interference. These results replicate the results of previous studies that examined the influence of practice in the Stroop task and align with a wide range of findings supporting the "law of practice" (e.g., Cohen et al., 1990; Davidson et al., 2010; Logan 1988). Interestingly, the decrease in response latency following training, which is believed to be a central marker of automatization, influences reading activity – a skill for which the pre-existing automatic character is hypothesized to induce interference in the Stroop task. Thus, more training can result in improved performance even in an extensively practiced skill.

Although we anticipated that the effects of training would be reflected in performance across conditions, namely that the delta in RTs between congruent and incongruent conditions would decrease, we found that the influence of training was restricted to performance *within* conditions. Despite a general reduction in RTs in both congruent and incongruent conditions, the congruency effect – characterized by slower RTs in incongruent trials compared to congruent trials – remained relatively stable throughout the four training sessions and during the 3-week period. These findings suggest that the underlying factors that drive the congruency effect are not affected by the amount of training or training in general.

Accordingly, the consistent gap in RTs between congruent and incongruent conditions can be explained by a systematic bias, insensitive to extensive practice. Preference for certain stimulus dimensions can influence attentional selection, irrespective of an individual's training level. In this case, more extensive training would not alter the relationship between incongruent and congruent stimuli pairs. The similar RTs in response to congruent and neutral stimuli suggest that such a systematic bias is unlikely to come from semantic facilitation (Parris et al., 2022).

The systematic bias observed may result from additional processing of the word dimension modulated by task conflict. This is consistent with race models of processing speed (Forrin & Macleod, 2017; Morton & Chambers, 1973), according to which interference in color identification on the incongruent condition occurs due to different processing speeds between the color and word dimensions, requiring that participants divide their attentional resources. Consequently, this finding points toward a mechanism of divided attention, where the systematic bias results from a competition between processes operating on different dimensions of the stimulus (Mordkoff & Yantis, 1991).

An alternative way to account for the consistent gap in RTs is that the improvements in performance across conditions reflect enhanced attentional control due to task switching (Monsell, 2003). Our task required flexibly shifting between different task rules on a trial-by-trial basis depending on the stimulus type (congruent, incongruent, or neutral). Recent research found that the ability to switch between stimulus-response mappings is a core component of general shifting ability (Von Bastian & Druey, 2017). This suggests that the training effects in our study could be attributed to an improved capacity to switch between response mappings for the different trial types. It was previously shown that predictable task sequences can facilitate advance preparation and reduce switch costs (Vandierendonck et al., 2010). Furthermore, a recent task-switching study found that participants showed a bias to select tasks in a way that created more modality-compatible than modality-incompatible mappings (Friedgen et al., 2024). These findings support the possibility that the effects of task-switching training improved performance across conditions.

While our findings demonstrate the effects of practice on reducing RTs in the Stroop task, it is important to consider whether these results generalize to other attentional control paradigms. Recent studies investigating the effects of training on the Flanker task, for example, found that a 3-week executive control training increased interference control in that task, as evidenced by reduced RTs in incompatible trials (Grützmann et al., 2022). Similarly, it has been reported that training in the Flanker task resulted in interference being reduced with training (Chen et al., 2013). Importantly, despite differences in the experimental designs, both studies observed reductions in interference in the Flanker task only within conditions (i.e., intra-condition). However, for the Stroop task, Chen et al. (2013) observed reductions in *both* inter- and intra-condition interference, contrasting with our present study in which only intra-condition reduction was observed.

Notably, both our study and that of Chen et al. (2013) found the most significant improvements in performance during the initial phases of the experimental procedure, between the first and second experimental sessions in our study, and between the first and second experimental blocks in Chen et al.’s study. This similarity suggests that performance improvements in attentional control tasks are characterized by rapid initial gains that diminish over subsequent iterations of the task. A key factor accounting for these differences is the fact that our study spanned multiple weeks, unlike Chen et al. (2013), where multiple blocks were conducted on the same day. This temporal difference suggests that interference may be sensitive to the distribution of practice over time. The frequency of repetitions could influence the reduction of informational conflict, implying that an alternative training regime might further reduce or even eliminate this effect in our paradigm.

The differences in interference reduction patterns between the Stroop and Flanker tasks may be explained by how task-relevant and task-irrelevant dimensions are processed. In the Stroop task, these dimensions may be processed in different pathways over time, while in the Flanker task both dimensions are likely processed in the same pathway. This difference could lead to distinct patterns of interference and training effects. Consequently, while practice effects are observed across and within attentional control tasks, their specific nature may vary depending on the task type and the temporal structure of the training.

One potential limitation of our study is the relatively small sample size used to detect the three-way interaction between group, congruency, and session. Although we based our power analysis on effect sizes reported in previous studies with similar designs (Jostmann & Koole, 2007; Milliken et al., 2012; Shichel & Tzelgov, 2018), the observed effects can be made even more robust. Consequently, the small sample size poses a challenge to the generalizability of the findings. Future studies should aim to replicate these findings with a larger sample to ensure the robustness of the observed effects.

In addition to the sample size, the temporal structure of our experimental design may have also influenced our findings. Our experimental design, in which participants trained for four non-equally spaced sessions over 3 weeks, allowed us to examine the influence of task conflict as the number of accumulated trials increased as well as changes that may result from decay following increasingly larger inter-session intervals (Altmann, 2002; García-Rueda et al., 2022; Hardt et al., 2013). This design also allowed to avoid any benefits to performance arising from spaced repetition.

The influence of practice on performance is known to be sensitive to repetitions that are expected to occur in constant temporal intervals (Cepeda et al., 2008; Melton, 1970; Tabibian et al., 2019). Performance improves more when the repetition of the task follows a consistent pattern of timing, compared to when the timing of repetitions is unpredictable (Logan, 1988). Nevertheless, since task conflict is assumed to play a strategic role in attentional allocation, a repetitive temporal structure might have been more conducive at the preparatory stage (i.e., prior to performing the task in a given session) than our experimental design allows for. A repetitive temporal structure might facilitate effective attentional allocation during the preparatory stage due to its familiarity. Future studies should examine the extent to which different temporal structures of task design bear on the role of task conflict in multi-session Stroop tasks.

Before concluding, we note that sleep-related learning mechanisms and circadian rhythms can significantly influence cognitive performance and task learning (Ruby et al., 2008; Sherman et al., 2015). In our study, the variability in timing of our second session, occurring either 24 or 48 h after the first one, may have introduced confounding factors, as sleep effects can differ between these two timeframes. Specifically, participants who had their second session 48 h later had an additional night of sleep that could have allowed for more sleep-dependent memory consolidation. Moreover, since running the sessions at different times of day can influence cognitive performance, the 24- versus 48-h gap may have led to more circadian variability between participants. Although by exposing participants to the same stimuli multiple times across different sessions and time points we may have reduced the impact of time-of-day performance variations on the overall results, future studies should aim to control for the precise temporal distribution of practice sessions over time to account for any sleep-related influence.

## Conclusions

Our study offers a novel way of examining the relationship between learning and task conflict, and its strength lies in integrating two approaches to evaluating the Stroop effect. The first approach examines how performance in the Stroop tasks changes over time with additional trials (where participants learn the task structure and demands). The second approach modulates the character and ratio of the relevant stimuli to separate the different types of conflict involved in the Stroop task (Levin & Tzelgov, 2014; Shichel & Tzelgov, 2018). To the best of our knowledge, our study is the first combination of these two approaches in a single Stroop study. Such integration allows the examination of how a major type of conflict implicated in the Stroop task – task conflict – influences behavior over multiple sessions. Because multiple sessions involve multiple loading of the task sets, alongside experience with task sets following the first session, our task provides evidence for the extent to which task conflict induces changes to attentional control on a more global scale. This has allowed us to examine the strategic use of attentional control rather than adjusting of attentional control on a local trial-to-trial basis. The inclusion of a temporal dimension in the Stroop task design is central to measuring the strategic aspects of task conflict.

## Data Availability

The data and materials for all experiments are available as specified in the Open Practices Statement.

## Footnotes

1 Henceforth, task conflict in the present study simply means a variation of the proportions of word to non-word stimuli (i.e., higher and lower proportions of words vs. non-words).

2 Several researchers (Entel & Tzelgov, 2018; Tzlegov et al., 1992; Tzelgov, 1997) have interpreted tasks with task conflict but without informational conflict as suggesting that there is no monitoring of automatic processes, namely such processes run to completion once started despite not being part of task demands. This interpretation has been based on the negative facilitation effect in which tasks with a higher proportion of neutrals exhibit larger negative facilitation.

3 While orthogonal planned contrasts do not typically require adjustment for multiple comparisons, as they are considered protected from inflated Type I error rates, it should be noted that given three contrasts, an adjustment for three comparisons would render this effect only marginally significant: Bonferroni-Holm correction – p = 0.055; Dunn correction – p = 0.054. This suggests caution in interpretation, indicating that the effect in the first session compared with the others might be considered a trend.

4 Thus, it can provide evidence supporting the alternative hypothesis over the null hypothesis. Typical evidence categories for BF values are: 1–3 = anecdotal, 3–10 = substantial, 10–30 = strong, 30–100 = very strong, >100 = decisive (Jeffreys, 1961).
