# Correction: Practice makes better? The influence of increased practice on task conflict in the Stroop task

**DOI:** 10.3758/s13421-025-01779-w

**Published:** 2025-08-25

**Authors:** Jonathan Najenson, Rut Zaks-Ohayon, Joseph Tzelgov, Nir Fresco

**Affiliations:** 1 Department of Philosophy, Ruhr-Universität Bochum, 44780 Bochum, Germany; 2 Mental Health Institute of Beer-Sheva, Ministry of Health, Beer-Sheva, Israel; 3 Faculty of Health Sciences, Ben-Gurion University of the Negev, 84105 Beer-Sheva, Israel; 4 Department of Cognitive and Brain Sciences, and School of Brain Sciences and Cognition, Ben-Gurion University of the Negev, 84105 Beer-Sheva, Israel

**Correction: Memory & Cognition**

10.3758/s13421-024-01677-7

The article “Practice makes better? The influence of increased practice on task conflict in the Stroop task”, written by Jonathan Najenson, Rut Zaks-Ohayon, Joseph Tzelgov, and Nir Fresco was originally published electronically on the publisher’s internet portal on 25 January 2025 without open access. With the author(s)’ decision to opt for open access, the copyright of the article changed on 29 July 2025 to © The Author(s) 2025 and the article is forthwith distributed under a Creative Commons Attribution 4.0 International License, which permits use, sharing, adaptation, distribution and reproduction in any medium or format, as long as you give appropriate credit to the original author(s) and the source, provide a link to the Creative Commons license, and indicate if changes were made. The images or other third party material in this article are included in the article's Creative Commons license, unless indicated otherwise in a credit line to the material. If material is not included in the article's Creative Commons license and your intended use is not permitted by statutory regulation or exceeds the permitted use, you will need to obtain permission directly from the copyright holder. To view a copy of this license, visit http://creativecommons.org/licenses/by/4.0/.

